# Glutathione–Allylsulfur Conjugates as Mesenchymal Stem Cells Stimulating Agents for Potential Applications in Tissue Repair

**DOI:** 10.3390/ijms21051638

**Published:** 2020-02-28

**Authors:** Emilia Di Giovanni, Silvia Buonvino, Ivano Amelio, Sonia Melino

**Affiliations:** 1 Department of Chemical Science and Technologies, University of Rome Tor Vergata, via della Ricerca Scientifica 1, 00133 Rome, Italy; emilia.digiovanni8@gmail.com (E.D.G.); s.buonvino@alice.it (S.B.); 2 MRC Toxicology Unit, University of Cambridge, Cambridge CB2 1QP, UK; ia348@mrc-tox.cam.ac.uk; 3 CIMER Center for Regenerative Medicine, University of Rome Tor Vergata, via Montpellier 1, 00166 Rome, Italy

**Keywords:** hydrogen sulfide, garlic, regenerative medicine, oxidative stress, MSCs, organosulfur compounds, cell migration, cell differentiation

## Abstract

The endogenous *gasotransmitter* H_2_S plays an important role in the central nervous, respiratory and cardiovascular systems. Accordingly, slow-releasing H_2_S donors are powerful tools for basic studies and innovative pharmaco-therapeutic agents for cardiovascular and neurodegenerative diseases. Nonetheless, the effects of H_2_S-releasing agents on the growth of stem cells have not been fully investigated. H_2_S preconditioning can enhance mesenchymal stem cell survival after post-ischaemic myocardial implantation; therefore, stem cell therapy combined with H_2_S may be relevant in cell-based therapy for regenerative medicine. Here, we studied the effects of slow-releasing H_2_S agents on the cell growth and differentiation of cardiac Lin^−^ Sca1^+^ human mesenchymal stem cells (cMSC) and on normal human dermal fibroblasts (NHDF). In particular, we investigated the effects of water-soluble GSH–garlic conjugates (GSGa) on cMSC compared to other H_2_S-releasing agents, such as Na_2_S and GYY4137. GSGa treatment of cMSC and NHDF increased their cell proliferation and migration in a concentration dependent manner with respect to the control. GSGa treatment promoted an upregulation of the expression of proteins involved in oxidative stress protection, cell–cell adhesion and commitment to differentiation. These results highlight the effects of H_2_S-natural donors as biochemical factors that promote MSC homing, increasing their safety profile and efficacy after transplantation, and the value of these donors in developing functional 3D-stem cell delivery systems for cardiac muscle tissue repair and regeneration.

## 1. Introduction

The *gasotransmitter* H_2_S is a physiological signalling molecule in mammalian cells that stimulates important molecular pathways [1,2,3]. Endogenous H_2_S is produced in tissues from l-cysteine by the activity of cystathionine γ–lyase (CSE), cystathionine β-synthase (CBS), thiosulfate:cyanide sulphurtransferase (TST, EC. 2.8.1.1; rhodanese) and 3-mercapto-piruvate sulfurtrasferase (3-MST) [4,5,6]. In the last decade slow H_2_S-releasing donors have been suggested as exogenous sources for therapeutic applications in cardiovascular [7,8,9], neurodegenerative [1,4,10] and gastrointestinal diseases [11,12]. One of most relevant problems in the H_2_S-based therapy is the identification of an appropriate posology and an accurate administration protocol of H_2_S donors, in order to avoid the high risk of overdosing. Therefore, slow H_2_S releasing agents, such as garlic derivatives, seem to exhibit the pharmacological features needed to generate H_2_S with a controlled rate and represent an interesting natural alternative for therapeutic applications. Organo-sulfur compounds (OSCs) derived from the garlic compound allicin, such as S-allylcysteine (SAC) diallyldisulfide (DADS) and diallyltrisulfide (DATS), have been recognized to have potential pharmacological properties, related to the H_2_S signaling pathway [13,14]. In particular, the allylsulfides DADS and DATS, which are the major components of oil-soluble garlic extract, are H_2_S slow-releasing donors. Their intracellular H_2_S-release mechanism requires the cooperation of reduced GSH, as elucidated by Kraus et al. [13]. In regards to the α carbon of a diallyl polysulphide, GSH acts as a nucleophilic substituent and the nucleophilic substitution leads to S-allyl glutathione and allyl perthiol [13]. By thiol/disulphide exchange with GSH, allyl perthiol can be transformed either into allyl glutathione disulphide (GSSH) and H_2_S, or into H_2_S_2_ and S-allyl glutathione through a nucleophilic substitution by GSH at the α-carbon. Finally, H_2_S_2_ can interact with GSH, resulting in GSSH and H_2_S. Therefore, polysulfides have recently been considered potential physiological mediators that are able to activate membrane channels, enzymes, and transcription factors by sulfhydration mechanism. The cytotoxicity of OSCs and H_2_S-donors in general likely depends on their concentration per cell and on their metabolic rate in the cells, which in turn depends on the cell type. The exogenous H_2_S can have pro- [15,16,17,18] or anti-apoptotic effects [19,20,21,22], depending on the individual cell phenotype and on the experimental settings used, such as the concentration of H_2_S. Previous studies suggest that garlic-derived OSCs selectively induce programmed cell death in neoplastic cells but not in their physiological counterparts or adult stem cells [23,24,25,26,27,28,29,30]. H_2_S is able, in fact, to improve cell survival in a cell-specific manner by activation of molecular signalling [31]. H_2_S represses programmed cell death and inflammation by downregulation of inflammatory cytokines, such as, for example, TNF-α, IL-1b, NF-kB, IL-8 and IL-6 [32,33,34,35]; furthermore, it regulates blood pressure–lowering, and exerts anti-nociceptive and cardioprotective effects due to the activation of cardiac extracellular signal-dependent-kinases, such as Akt pathways and K_ATP_ channels [36,37].

To assess the effects of H_2_S-donors with antitumor properties on adult stem cells, in this study, water-soluble glutathione-garlic extract (GSGa) was produced using the protocol previously described [16,38], and it was used for treatment of human adult stem cells. GSGa is a particular extract rich in glutathione-conjugates with pro-apoptotic properties on cancer cell lines and the ability to promote their G2/M phase cell cycle arrest [16]. The data herein presented demonstrate that, in contrast with the effects on tumor cells, GSGa treatment of cardiac Lin^−^ Sca-1^+^ human mesenchymal stem cells (hereinafter, cMSC) improves their viability, proliferation and migration rate, without affecting their plasticity. The effects of the treatment on cMSC were also compared with other H_2_S-donors, such as Na_2_S and GYY4137. Our previous studies performed on other H_2_S releasing systems (nanoemulsions, hydrogels and nanofibers) showed that the H_2_S release improves the proliferation of cMSC, as well as of normal human dermal fibroblasts (NHDF), and increases the expression of proteins related to cell–cell interaction, such as connexin 43, and cell survival under oxidative stress [38,39,40]. H_2_S-donors, in fact, display relevant antioxidant properties; they can either act as reducing agents/scavengers by directly reacting with ROS species or rescue the cells from oxidative stress by promoting glutathione production, which is the most abundant and potent intracellular antioxidant species [41]. Exogenous H_2_S can protect primary rat cortical neurons from oxidative stress, so it can be a powerful neuroprotective agent due to its cytoprotective, anti-inflammatory, antioxidant and anti-apoptotic properties [41,42,43,44]. Furthermore, recent studies demonstrate that H_2_S preconditioning protects bone marrow-derived mesenchymal stem cells (BMSCs) from hypoxia, and biologically-active factors released by conditioned-H_2_S BMSCs provide protection of neurons exposed to ischaemic conditions [45]. On these bases, we investigated the antioxidant properties of GSGa, demonstrating its ability to inhibit cell death induced by ROS damage or CoCl_2_ in cMSC and normal human dermal fibroblasts (NHDF). These properties were related to an upregulation of antiapoptotic and antioxidant proteins, such as Bcl2, NAD(P)H quinone oxidoreductase 1 (NQO1) and thioredoxin (Trx). Furthermore, a prolonged treatment of cMSC with GSGa allowed us to obtain a selected cMSC line, named GcMSC, which showed an increased proliferation and migration rate as well as resistance to oxidative stress, while preserving the stem cell multi-potency. Therefore, these results suggest a potential use of prolonged treatment with H_2_S-donors for the optimization of adult stem cells for tissue engineering and repair.

## 2. Results

### 2.1. H_2_S-Donors Promote Proliferation and Migration of cMSC

**Figure 1:**
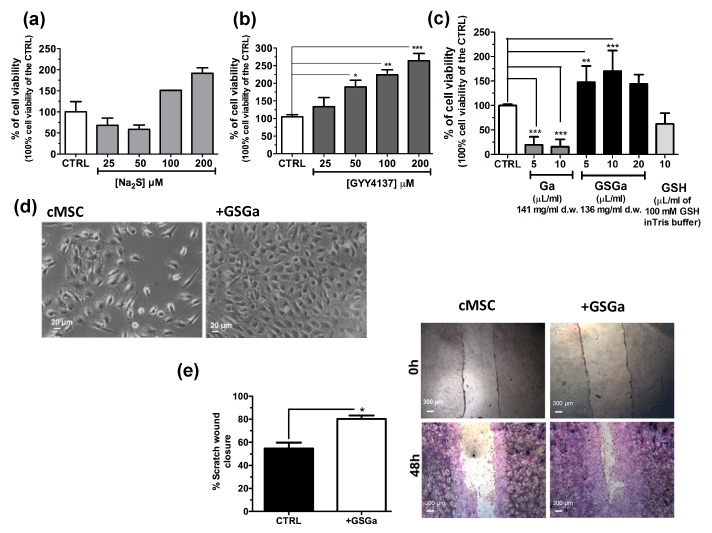
Effects of H_2_S slow-releasing agents on cell proliferation and migration of cMSC. Cell proliferation of cMSC (5 × 10^3^ cells/cm^2^) after 3 days of treatment at different concentrations of (**a**) Na_2_S (25, 50, 100 and 200 μM) and (**b**) GYY4137 (25, 50, 100 and 200 μM); (**c)** cell proliferation of cMSC treated for 3 days with 5 and 10 μL of Ga (141 mg/mL d.w) and 5, 10 and 20 μL of GSGa (136 mg/mL d.w). (**d**) Bright field micrographs of cMSC cultured for 3 days in the presence and in the absence of 136 μg/mL of GSGa. (**e**) Scratch wound healing assay on cMSC cultured in the absence (cMSC) or in the presence of 680 μg/mL of GSGa (+GSGa). Micrographs in the upper and lower panels were taken immediately after the scratching (0 h) and after 48 h (48 h) respectively; cells shown in the lower panels were stained with crystal violet dye. Quantification of wound closure was calculated by three independent experiments. Error bar indicates S.D.; *n* = 3 or 5. Statistical significance is shown as * *p* value ≤ 0.05, ** *p* value ≤ 0.01, *** *p* value ≤ 0.005.

The effects of H_2_S on stem cells have not been fully investigated. To further study the effect of H_2_S donors on human adult stem cells, Na_2_S, GYY4137 and water-soluble garlic extracts (GSGa) were used for the treatment of cMSC. In Figure 1, the effects of H_2_S releasing agents on the cell proliferation of cMSC are shown. Cell viability was analyzed after three days of treatments with different H_2_S-donors and, in general, an increase of cMSC proliferation in a concentration-dependent manner was observed with respect to the control, especially after treatment with GYY4137 and GSGa (Figure 1b,c). Notably, Na_2_S treatment induced a decrease in proliferation at 25 and 50 μM concentrations, probably due to a direct cytotoxic effect for the high concentrations of H_2_S. By contrast, at higher H_2_S concentrations, 100 and 200 μM, an increase in cell proliferation was observed after 3 days of growth (Figure 1a). 

Both GYY4237 and GSGa induced an increase in the cell proliferation in a concentration dependent manner. After three days of treatment with 680 μg/mL of GSGa, a statistically significant increase of up to 170.3 ± 41.99% of cell proliferation with respect to the control was observed (Figure 1c). By contrast, the treatments with both water-soluble garlic extracts obtained without reaction with GSH (Ga) and GSH, at similar concentrations used for the GSGa treatment, did not induce an increase in cell viability, demonstrating that the relevant role on the induction of the stem cell proliferation by GSGa treatment is due to the presence of glutathionyl-conjugates. A cell proliferation increase due to the GSGa treatment was also confirmed by microscopy analysis, as shown by micrographs of the cells after three days culture in the presence or in the absence of 136 μg/mL of GSGa (Figure 1d). The proliferation and migration were also assessed by scratch test (Figure 1e); the cell cultures after 48 h in the presence of GSGa showed a closer scratch than in the absence, probably due to concomitant increase in both cell migration and proliferation.

### 2.2. Antioxidant Properties of GSGa and Increase in cMSC Survival under a Chemical Hypoxia-Mimicking Agent

**Figure 2:**
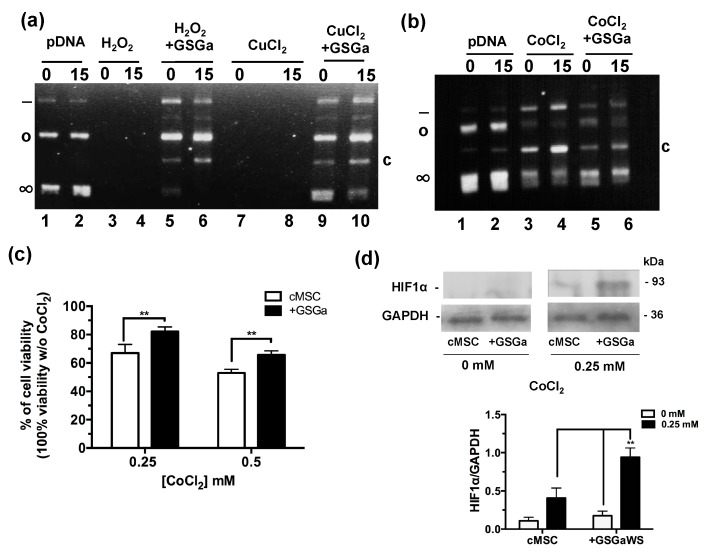
Antioxidant properties of GSGa. (**a**) Inhibition of pDNA cleavage by GSGa; 0.5 μg of pDNA after addition of H_2_O_2_ (100 μM) or CuCl_2_ (100 μM) and ascorbic acid (10 mM) in 20 mM Tris HCl, pH 7.4, buffer after 0 (lane 1) or 15 min (lane 2) of incubation at 37 °C in the absence (lane 3, 4, 7 and 8) or in the presence (lane 5, 6, 9 and 10) of 10.8 μg/μL of GSGa. (**b**) 0.5 μg of pDNA after addition of CoCl_2_ (100 μM) and ascorbic acid (10 mM) in 20 mM Tris HCl, pH 7.4, buffer after 0 (lane 1) and 15′ (lane 2) incubation at 37 °C in the absence (lane 3 and 4) or in the presence (lane 5 and 6) of 10.8 μg/μL of GSGa. Note: (∞), (o), (-) and (c) are respectively the supercoiled, circular, linear and cleaved pDNA forms. (**c**) Cell viability of cMSC (by MTT assay) after 40 h of treatment with 0, 0.25 and 0.5 mM of CoCl_2_ in the presence (+GSGa) or in the absence (cMSC) of 680 μg/mL of GSGa. The integral images of the gels are in reported in the Supplementary Materials (Figure S3). (**d**) western blot and densitometric analysis of HIF-1α expression in cMSC after exposure to CoCl_2_ in the presence or in the absence of GSGa in the culture medium. Error bar indicates S.D. Blots are a representative experiment of three independent experiments. ** *p* value < 0.01 (one-way ANOVA test).

The antioxidant properties of the GSGa was tested using a pDNA damage assay based on assessing the cleavage effect of oxidant species such as H_2_O_2_ or Cu^2+^ and Co^2+^ ions in the presence of ascorbic acid (Figure 2a,b). The presence of GSGa at 10.8 μg/μL inhibited the pDNA cleavage obtained in both cases: after incubation with 100 μM of H_2_O_2_ and after incubation with 100 μM of Cu^2+^ and 10 mM ascorbic acid. The pDNA cleavage was also reduced in the presence of 100 μM of CoCl_2_ and 10 mM ascorbic acid by addition of GSGa, as shown by the presence of the supercoiled form of the pDNA and the reduced presence of a cleaved form after treatment with CoCl_2_ (Figure 2b). The effects of CoCl_2_ on the cMSC viability were also studied in the presence and in the absence (see also Figure S2 in Supplementary Materials) of 680 μg/mL of GSGa in the cell culture medium (Figure 2c), showing a protective anti-hypoxic effect of the GSGa, in turn resulting in an increase of 15% in cell survival in the presence of 0.25 mM of CoCl_2_. An increase in the expression of HIF-1α was observed under CoCl_2_ treatment in the presence of GSGa (Figure 2d).

Therefore, the cytoprotective effect of GSGa against CoCl_2_ could promote the expression of hypoxia-responsive element (HRE)-controlled genes by reduction of HIF-1α subunit degradation. 

### 2.3. GSGa Improves Cell Survival under Oxidative Stress

**Figure 3:**
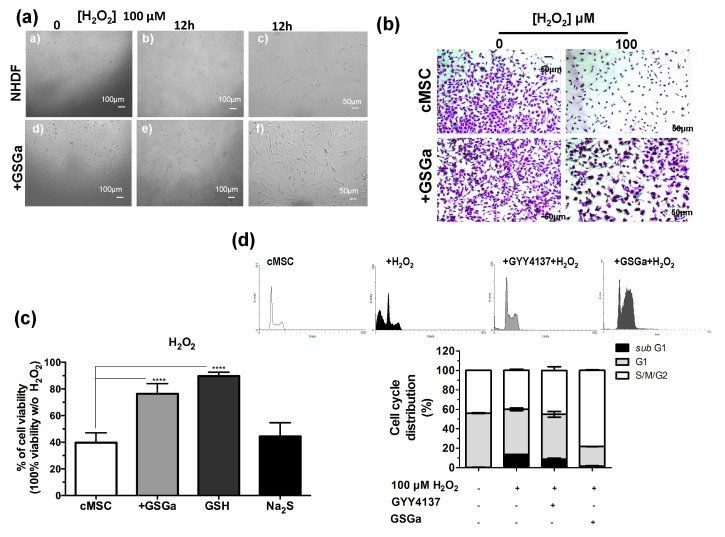
Antioxidant effect of GSGa on cells. (**a**) Micrographs of NHDF after 0 and 12 h of treatment with H_2_O_2_ (100 μM), with (+GSGa) or without (NHDF) the addition of 680 μg/mL GSGa (see also Figure S5); **(b**) micrographs of cMSC (CTRL) after 24 h of treatment with H_2_O_2_ (100 μM), with or without the addition of 680 μg/mL of GSGa. Cells were dyed with crystal violet; (**c**) Cell viability of cMSCs after 24 h of growth in the presence of H_2_O_2_ (100 μM) (cMSC) (as control) and in the presence of 680 μg/mL of GSGa (+GSGa) or Na_2_S (95 μM) or GSH (100 mM); (**d**) FACS cell cycle analysis of cMSC cultured for 24 h in the presence of H_2_O_2_ (100 μM) and with addition of either GYY4137 (80 mM) or GSGa (680 μg/mL). Error bar indicates S.D. Experiments were performed in three or five biological replicas. **** *p* valule ≤ 0.0001 (one-way ANOVA).

H_2_S is a physiological mediator that limits inflammation and free radical damage [46] and is able to upregulate ARE-gene transcription [47]. In Figure 3, the protective effects of the pretreatment with GSGa from the oxidative damage induced by H_2_O_2_ on NHDF and cMSC cultures are shown. Figure 3a shows the micrographs of NHDF after 12 h of H_2_O_2_ exposure and pretreating with GSGa. After three days of growth in the presence (GSGa) or in the absence (NHDF) of 680 μg/mL of GSGa, the cells were re-seeded, and after adhesion (6 h from seeding), H_2_O_2_ was added to the cell culture medium at a final concentration of 100 μM. Pretreatment with GSGa induced major resistance to the oxidative stress due to presence of H_2_O_2_, as was observable by the presence of a larger number of the cells in the well (Figure 3a,b).

Moreover, the presence of GSGa in the medium significantly prevented H_2_O_2_-induced cell death of cMSC (Figure 3b). To determine whether GSGa treatment of cMSC could protect from oxidative damage, cMSC were treated with 100 μM H_2_O_2_ for 24 h either in the presence or in the absence of 680 μg/mL of GSGa (Figure 3c). An increase of 36.7% cell survival in the presence of GSGa with respect to its absence was observed. Furthermore, the effect due to the presence of GSH and Na_2_S in the medium was also analyzed, resulting in cell viability of 89.7%, compared to 44.4% cell viability without H_2_O_2_. The FACS cell cycle profiles after treatment confirmed the increased percentage of cMSC in the *subG_1_*-phase after treatment with H_2_O_2_, which was not observable in the presence of GSGa, confirming the antioxidant properties of the glutathione-conjugate extract. Moreover, in the presence of a known H_2_S-donor, such as GYY4137, the same decrease in the *subG_1_*-phase was not detected (Figure 3d).

### 2.4. GSGa Increases the Expression of Pro-Cell Survival and Differentiation-Associated Markers

**Figure 4:**
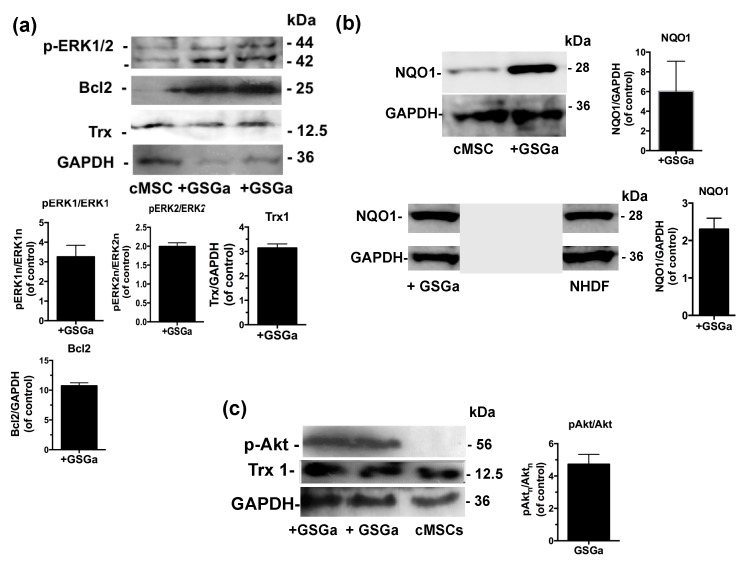
Effects of the GSGa on the protein expression. (**a**) Representative western blot analysis of the expression of p-ERK1/2, Bcl2, Trx1 and GAPDH in cMSC cultured for 3 days in the presence (+GSGa) or in the absence (cMSC) of 680 μg/mL of GSGa; (**b**) Representative western blot analysis of the expression of NQO1, GAPDH in both cMSCs and NHDFs cultured for 3 days in the presence (+GSGa) or in the absence (MSC or NHDF) of 680 μg/mL of GSGa, The integral figure of the NHDFs blot and the original western blot of MSC for NQO1 expression are shown in the Supplementary Materials (Figures S9 and S10); (**c**) Representative western blot analysis of the expression of p(Ser 473)Akt in cMSC after 3 days of treatment with (+GSGa) or without (cMSC) GSGa, GAPDH expression was used as normalization control. Error bar indicates s.e.m or S.D. Experiments were performed in three or five biological replicas (one-tailed Student’s t-test).

The molecular mechanisms underlying GSGa’s cytoprotective effect on both NHDF and cMSC were investigated using western blot analysis of the cellular extract after 3 days of treatment with GSGa (680 μg/mL). The expression of the proteins involved in the cellular redox system, such as thioredoxin 1 (Trx1) and NQO1, and the anti-apoptotic protein Bcl2, were substantially increased after treatment with GSGa (Figure 4a,b). In particular, Bcl2 was increased up to 10 times compared to control non-treated cMSC (Figure 4a). 

Furthermore, to determine the potential pathways involved in the GSGa-mediated pro-cell survival effect, the activation of extracellular signal-regulated kinases ERK1/2 and Akt, also known as protein kinase B, was observed in cMSC after GSGa treatment (Figure 4a,c). An increased activation of ERK1/2 (p-ERK1/2) was observed in treated cMSC compared to control (Figure 4a), therefore ERK1/2 could be responsible for the pro-survival and anti-apoptotic effect of GSGa. After three days of cell culture in the presence of 680 μg/mL of GSGa (Figure 4c), the intracellular level of p(Ser 473)-Akt in cMSC was increased.

**Figure 5:**
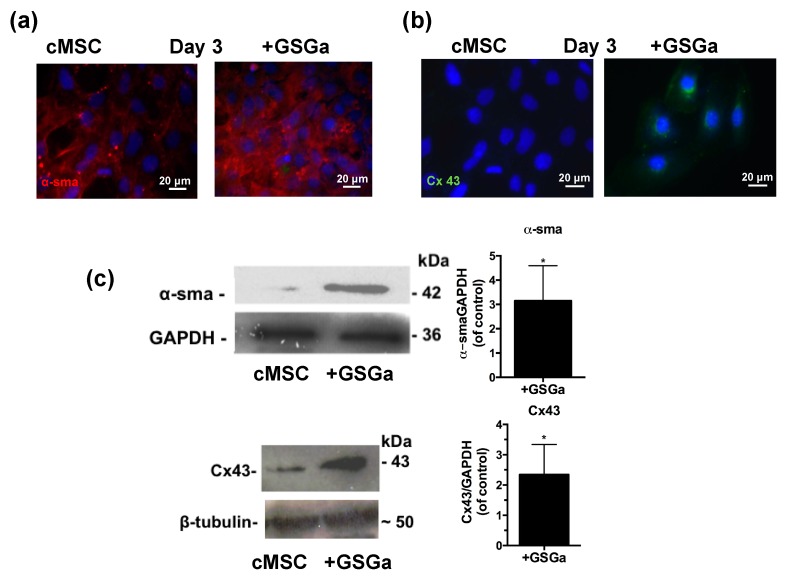
GSGa increases the expression of cardiac phenotype markers. Fluorescent micrographs of 5 × 10^3^ cells/cm^2^ of cMSC cultured for 3 days in the presence (GSGa) and in the absence of 680 μg/mL of GSGa, (**a**) α-SMA expression is shown in red and (**b**) Cx43 is stained in green; (**c**) western blotting and densitometric analysis of the protein expression in cMSC after 3 days of treatment with 680 μg/mL of GSGa. The expression of the proliferation marker and the proteins Cx43 and α-SMA was significantly upregulated after treatment compared to the control. Images are representative of five independent experiments. Error bar indicates S.D. Experiments were performed as three biological replicas. * *p* value ≤ 0.05 (one-tailed Student’s t-test).

The expression of proteins involved in the commitment of the cardiac muscle phenotype differentiation was also assessed by fluorescence microscopy and western blot analysis. Figure 5a,b show the fluorescence micrographs of cMSC after 3 days of growth in the presence and in the absence of 680 μg/mL of GSGa. An increased expression of the proteins α-smooth muscle actin (α-SMA) and connexin 43 (Cx43) were observed in the cMSCs cultured in the presence of GSGa and the increased expression was maintained over time after 6 or 7 days of treatment (Figure S7 in Supplementary Materials). As shown in Figure 5c, treatment of cMSC with 680 μg/mL of GSGa led to a significant increase of the expression of α-SMA and Cx43 proteins.

### 2.5. Prolonged Treatment with GSGa Stimulates Cell Proliferation, Resistance to Oxidative Stress and Migration of cMSC

**Figure 6:**
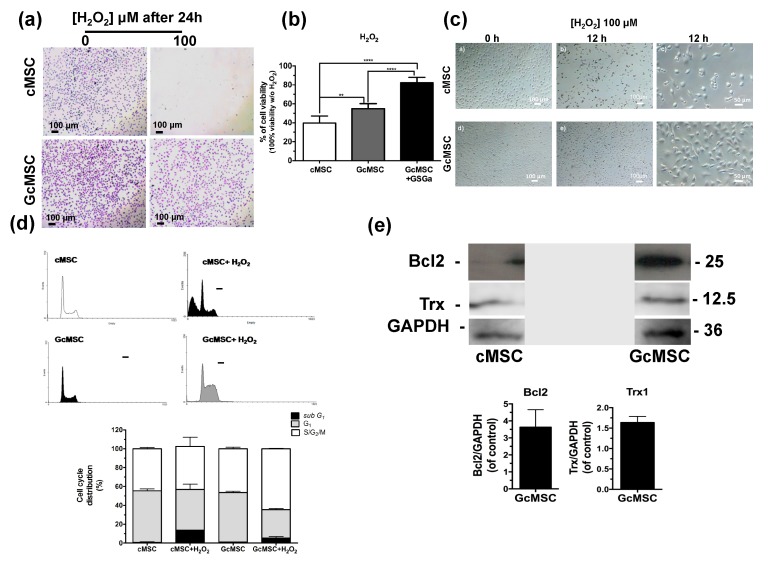
Prolonged GSGa treatment for selection of oxidation-resistant cMSC line. (**a**) Micrographs of cMSC and GcMSC seeded at 1 × 10^4^ cells/cm^2^ of density, after 24 h from seeding in the presence or absence of H_2_O_2_ (100 μM). Scale bars are 100 μm. Cells were stained using crystal violet. (**b**) Cell viability of control (cMSC), GcMSC (GcMSC) and of GcMSC in the presence also of 680 μg/mL of GSGa (GcMSC+GSGa) after 24 h of treatment with H_2_O_2_ (100 μM). The data were obtained by five independent experiments and analyzed using ANOVA one-way test. (**c**) Optical micrographs of cMSC and GcMSC (1 × 10^4^ cells/cm^2^ of density) at 0 or 12 h of treatment with H_2_O_2_ (100 μM). Scale bars are 100 and 50 µm; (**d**) FACS profiles (on the top) and histograms of the cell cycle distribution (on the bottom) of cMSC (as control) and GcMSC cultured for 12 h in the presence or in the absence of H_2_O_2_ (100 μM). (**e**) Representative western blot analysis of the expression of Bcl2, Trx1 in cMSC and GcMSC. The original figure is shown in the Supplementary Materials Figure S9. Experiments were performed as three biological replicas. Error bar indicates S.D. ^**^ *p* value ≤ 0.01; **** *p* value ≤ 0.0001 (one-way ANOVA).

Thanks to the cytoprotective properties of H_2_S-donors, the preconditioning of stem cells before their transplantation has recently gained attention. On this basis, we performed a prolonged pretreatment of the cells using a low concentration of GSGa with the aim to select cMSC with more resistant features to the oxidative stress compared to non-treated cells. In detail, cMSC were cultured for one month in the presence of 140 μg/mL of GSGa and the selected line was named GcMSC. The resistance to oxidative stress was tested by exposure of cMSC and GcMSC to 100 µM H_2_O_2_ for 24 h. Figure 6a shows the representative optical micrographs of the cells after H_2_O_2_ treatment, where it is evident that the GcMSCs had higher cell density and improved morphology compared to cMSC after treatment. 

This result was also confirmed by a WST-1 viability assay (Figure 6b). GcMSC showed a survival of up to 15 ± 5.3% higher than the cMSC used as control. Notably, further addition of GSGa to the medium of the GcMSC culture, led to a survival of 42.5 ± 5.7%. This last value was higher than that obtained with only the addition of GSGa (36.7% ± 7.67, Figure 3c) without preconditioning.

**Figure 7:**
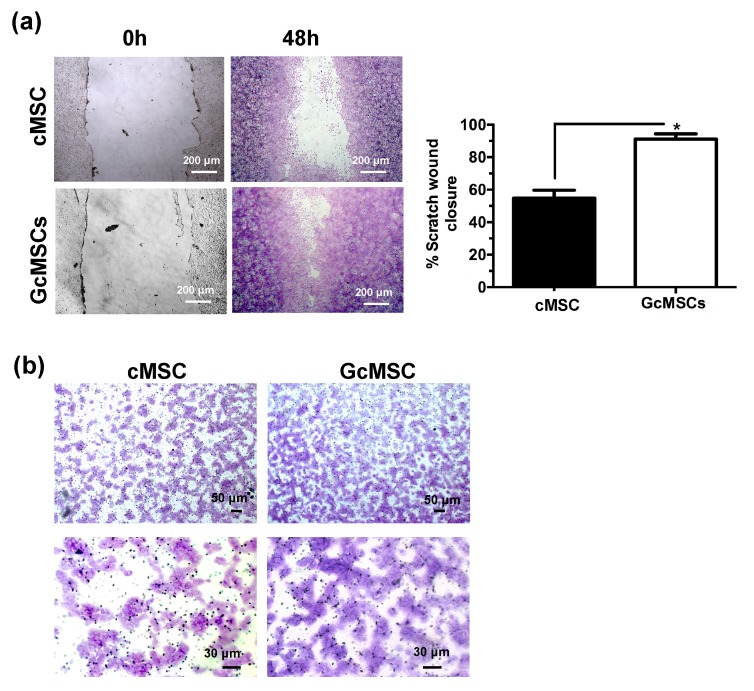
Prolonged GSGa treatment improves cMSC migration. (**a**) Scratch wound healing assay of GcMSC and cMSC; micrographs in the upper and lower panels were taken immediately after the scratching (0 h) and after 48 h of growth respectively. The quantification of the wounded area invaded was calculated by two independent experiments. (**b**) Micrographs of a 6 h trans-well migration assay of GcMSC and control cMSC. Cells were stained with crystal violet dye. Scale bars are 200 μm in panel A, and 50 μm (upper) and 30 μm (lower) in panel B, respectively. * *p* value ≤ 0.05 (one-tailed Student’s t-test). Error bar indicates s.e.m.

GcMSC were more resistant than cMSC to the oxidative damage by the cellular density after 12 h of treatment with H_2_O_2_ (Figure 6d). This finding was also confirmed by the cell cycle analysis, which showed that the percentage of the GcMSC population in the *subG_1_*-phase was half that of cMSC. This increased resistance to the oxidative stress was also in agreement with higher levels of expression of proteins associated with pro-survival Bcl2 and Trx1 in GcMSC compared to those in cMSC (Figure 6e and Figure S6b in Supplementary Materials). The cell migration ability was also tested using both the percentage of scratch wound closure after 48 h, shown in Figure 7a, and the trans-well migration through the membrane after 6 h. In both cases, the GcMSC migration was significantly higher than that of cMSC.

### 2.6. Prolonged Treatment with GSGa Does Not Affect the Mesenchymal Stem Cell Plasticity

**Figure 8:**
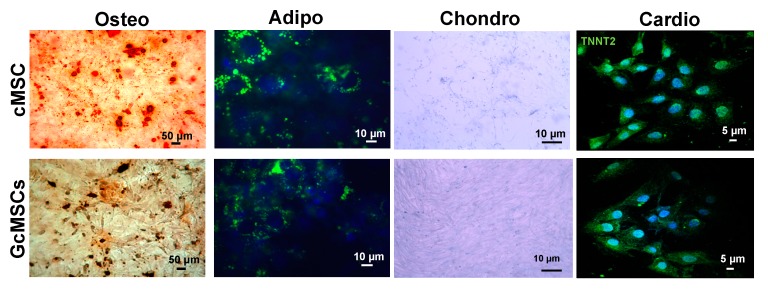
Multipotency of cMSC and GcMSCs. Chondrogenic, adipogenic and osteogenic differentiation of cMSC and GcMSC (cMSC pretreated with 136 μg/mL of GSGa for one month). The cells were stained using Alizarin red S, AdipoRed and Alcian blue dyes for osteogenic, adipogenic and chondrogenic differentiation, respectively. For cardiogenic differentiation, cells were stained using an anti-TNNT2 and an Alexa Fluor^®®^ 488 donkey anti-mouse secondary antibody. Scale bars are 5, 10, 50 μm as indicated. Experiments were performed as three biological replicas.

To assess the multipotency of the GcMSC, they were subjected to different differentiation protocols. As shown in Figure 8, GcMSC were able to differentiate into different cell types: osteoblasts, adipocytes, chondrocytes and cardiocytes. In particular, the cardiogenic differentiation was assessed by the expression of human troponin T2 (TNNT2). The microscopic analyses of the cells did not show any relevant differences between the cell plasticity of GcMSC and cMSC. 

### 2.7. GSGa Treatment of cMSCs Modulates the Expression of Genes Related to the Cardiovascular Disease and Detoxification Enzymes

**Figure 9:**
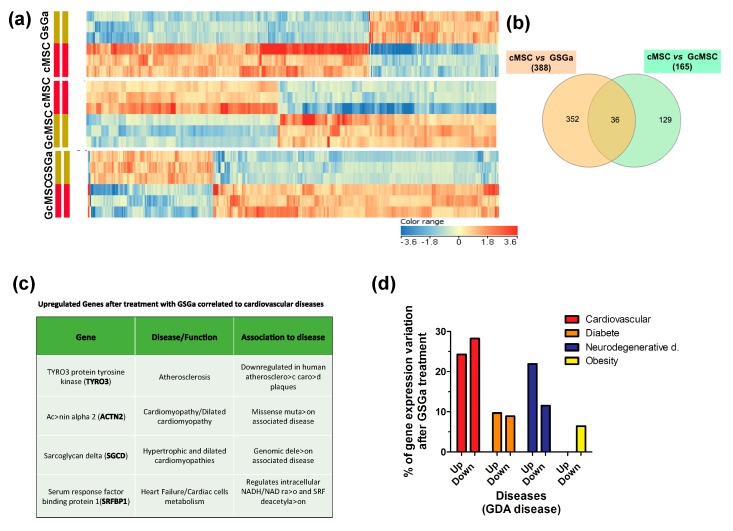
Transcriptional profiling of cMSC following GSGa treatment. (**a**) Heat-maps show significantly upregulated and downregulated genes in cMSC following prolonged (GcMSC/cMSC) or 3-day (GSGa/cMSC) GSGa treatment and those in GSGa with respect to GcMSCS (GcMSC/GSGa). Heat-maps were generated by hierarchical clustering of genes accordingly to the fold change. Cut-off *p* value ≤ 0.05; Cut off fold change 1.5. (**b**) Venn diagram depicts overlap of differentially regulated genes after 3 days (GSGa) and 30 days (GcMSC) of treatment. The diagram has been generated by using InteractiVenn webtool (http://www.interactivenn.net) inputting the probe name lists from the gene array data. (**c**) Selection of significantly upregulated genes following GSGa treatment whose function was associated with cardiovascular diseases. (**d**) Histograms show gene expression variation (upregulation: up; downregulation: down) after GSGa treatment (GSGa) of genes associated with the diseases indicated in the legend.

To determine the signaling pathways modulated by the treatment with GSGa, the global transcriptional profile (50,599 genes) of cMSCs was analyzed by gene microarray analysis. In total, 388 and 165 genes showed modulated expression in the cMSCs treated for 3 days with GSGa and in the GcMSCs, respectively, compared with the control. In detail, 266 genes were downregulated and 122 were upregulated after GSGa treatment with respect to the control, while 77 genes were upregulated and 88 downregulated in GcMSC with respect to cMSC. Figure 9a shows the respective heat-maps. Volcano plots from microarray analysis are shown in Figure S8 (in Supplementary Materials). Venn diagram (see Figure 9b) of cMSC vs. GSGa and cMSC vs. GcMSC shows that 36 genes were regulated in both acute and prolonged preconditioning. Interestingly, the transcription of genes involved in the metabolism of xenobiotics and the MAPK signaling pathway, such as cytocrome P450 and myocyte enhancer factor 2C (MEF2C), was changed, after both the preconditioning treatments (see Table S1 in Supplementary Materials). However, these results must be confirmed by protein expression analysis.

Among the proteins upregulated after treatment, four proteins were found to be either downregulated or not expressed at all in certain cardiovascular diseases. In particular, the protein tyrosine-protein kinase receptor (TYRO3) was found to be upregulated in treated cMSC, while its expression is reported to be downregulated in atherosclerotic carotid plaque [48]. Actinin alpha 2 (ACTN2) was also upregulated upon treatment with GSGa, while it has been reported to be downregulated in cardiomyopathy/dilated cardiomyopathy [49]. This protein is expressed in skeletal and cardiac muscle tissue and it serves as a bridge for anchoring myofibrillar actin thin filaments and titin to Z-discs. Finally, two more proteins were found to be upregulated upon treatment: sarcoglycan delta (SGCD) and serum response factor binding protein 1 (SRFBP1), usually downregulated or mutated in hypertrophic and dilated cardiomyopathies and during heart failure, respectively [50,51].

The analysis, performed using DAVID 6.8 program (https://david-d.ncifcrf.gov/home.jsp), of the functions of the genes both up and downregulated in cells after GSGa treatment with respect to the control showed that 52.5% of the genes whose expression was altered upon the treatment were linked to the cardiovascular system/cardiovascular diseases (approximately 24.3% were upregulated and the remaining 28.2% were downregulated), 33.4% of the altered genes were linked to neurodegenerative diseases (21.9% upregulated, 11.5% downregulated), and 18.6% were associated with diabetes (9.7% upregulated, 8.9% downregulated) (Figure 9c). The remaining 6.4% were associated with obesity and were all downregulated (Figure 9c).

## 3. Discussion

The water-soluble garlic extract, named GSGa, obtained by conjugation of garlic OSCs with glutathione was here investigated for its antioxidant properties and its ability to induce cell proliferation and migration in adult stem cells. 

Recently, in vivo and in vitro studies have shown that NaHS is able to enhance the survival of bone marrow-derived mesenchymal stem cells (BMSC) upon hypoxia-ischaemic conditions [45]. Our results show an increase of the cMSC proliferation in a concentration-dependent manner with respect to the control after treatment with GYY4137 and GSGa. These data are in agreement with previous results demonstrating the ability of H_2_S to promote neural stem cell proliferation and differentiation and to protect against hypoxia-induced decreases in hippocampal neurogenesis [52].

The cytotoxic effect observed at 50 μM of Na_2_S could be compatible with a very fast H_2_S-release, while at higher concentrations this initial cytotoxic effect was compensated by a positive effect on the cell growth of a slow H_2_S release due to the protein S-sulfhydration. As already described, the cellular or tissue response may be influenced by the manner in which cells and tissues are exposed to H_2_S [53]. Both Na_2_S and NaHS instantaneously generate H_2_S and are very short-lived compounds, indeed they are not ideal H_2_S-donors for studying the physiology, whereas enzymatic or GSH-derived H_2_S synthesis is considerably slower, over a much longer time [13,54,55,56]. The GSGa was also able to improve the stem cell migration as demonstrated by the scratch assays where the cell cultures after 48 h in the presence of GSGa showed a closer scratch than in the absence. This effect was probably due to concomitant increase in both cell migration and proliferation.

The antioxidant properties of the GSGa was also tested using a pDNA damage assay based on assessing the cleavage effect of oxidant species such as H_2_O_2_ or Cu^2+^ and Co^2+^ ions in the presence of ascorbic acid. The presence of GSGa inhibited the pDNA cleavage obtained in both H_2_O_2_, CuCl_2_ and CoCl_2_. GSGa showed a protective anti-hypoxic effect on the cMSC, leading to an increase of 15% in cell survival with increased expression of HIF-1α. The HIF-1α subunit of the heterodimeric transcription factor HIF is continuously synthesized, hydroxylated and degraded through the ubiquitin–proteasome system [57]. Under hypoxic or hypoxic-mimic conditions, such as after treatment with CoCl_2,_ a chemical hypoxia-mimicking agent, the prolyl-hydroxylase activity is inhibited and consequently HIF-1α is stabilized for translocation into the nucleus and dimerization with HIF-1β [58]. Active HIF-1 regulates the expression of genes involved in the anti-hypoxic/oxidant cellular response. Target genes of HIF-1 are involved in different biological pathways, such as energy metabolism, angiogenic signaling, growth, apoptosis, and cell migration [59]. The cytoprotective effect of GSGa against CoCl_2_, similarly to other H_2_S-donors [60], could reduce HIF-1α subunit degradation, promoting the expression of hypoxia-responsive element (HRE)-controlled genes. 

During the last decade, it has been demonstrated that H_2_S is a physiological mediator that limits inflammation and free radical damage [46] by reacting with multiple oxidant stressors including peroxynitrite [57], superoxide radical anion [61], and hydrogen peroxide [62]. Moreover, H_2_S is able to upregulate ARE-genes transcription [47] and also produce glutathione persulfide (GSSH) in mitochondria [63,64,65], a more efficient H_2_O_2_ -scavenging molecule than GSH. The pretreatment with GSGa of both NHDF and cMSC cultures led to an increase in cell survival from the oxidative damage induced by H_2_O_2_.

The presence of GSGa led to an increased percentage of cells in the *S/M/G_2_*-phase, which could be related to both an increase of *S*-phase or a partial cell-cycle arrest in *G_2_* phase. This increased rate of MSCs in the *S/M/G_2_*-phase was not observed in the presence of GSGa, without the addition of H_2_O_2_ (see Figure S4 in Supplementary Materials). Therefore, the GSGa treatment could have both a direct protective effect against oxidative species, as demonstrated using the pDNA degradation assay, and an indirect effect in which it elicits a cellular antioxidant response by inducing ARE-controlled gene expression.

The molecular mechanisms underlying GSGa’s cytoprotective effect on both NHDF and cMSC were here investigated using western blot analysis.

**Figure 10:**
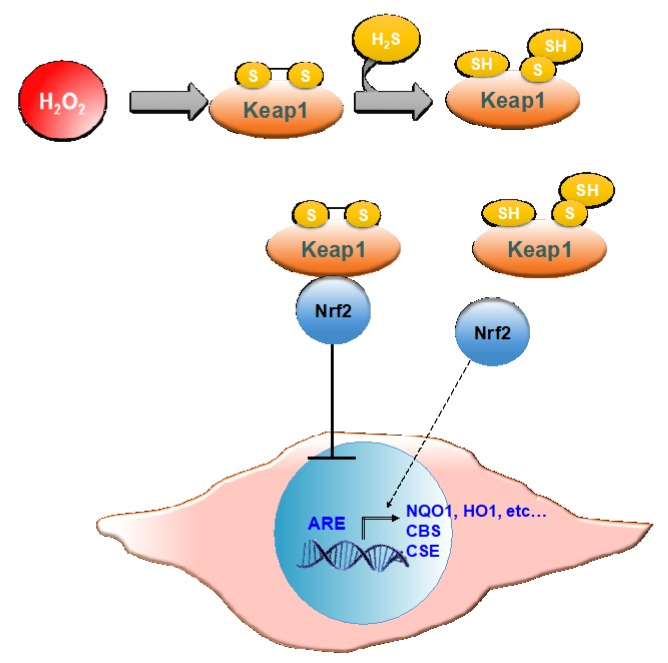
Schematic representation of Nrf-2 activation of ARE by H_2_S. Keap 1, Kelch-like ECH-associated protein 1; Nrf2, nuclear erythroid factor 2-related factor 2; ARE, antioxidant response elements; NQO1, NAD(P)H quinone oxidoreductase 1; HO1, heme oxygenase 1; CBS, cystathionine β-synthase; CSE, cystathionine γ–lyase.

In both cMSC and NHDF, the treatment with GSGa also led to an increased expression of NQO1, and these results are also in agreement with previous findings [45]. NQO1 is one of the target proteins of the transcription factor Nrf2, involved in the antioxidant response of mammalian cells. NQO1′s primary activity is the 2-electron reduction of endogenous and exogenous quinones to their corresponding hydroquinones through the use of either NADH or NADPH as the hydride donors, thus preventing the formation of radical species [66]. NQO1′s antioxidant effects also correlate with its role in preserving a reduced pool of endogenous antioxidant molecules and stabilizing the tumor suppressor protein p53 [67]. The activity of antioxidant enzymes is regulated in the cell by the Nrf2-antioxidant response element (ARE) pathway. Nrf2 is a transcription factor normally found in the cytoplasm bound to the Kelch-like ECH-associated protein 1 (Keap1), which acts as a substrate adaptor protein for the Cullin3 (Cul3)-containing E3-ligase complex. The interaction between the aforementioned complex and Keap1 leads to Nrf2 ubiquitination and, eventually, to its degradation [68]. During oxidative stress, Keap1 undergoes structural modifications that result in the exposure of lysine residues, which become targets for ubiquitination and subsequent degradation [69]. Similarly, H_2_S is able to induce the dissociation between Nrf2 and Keap1 through the sulfhydration of the Cys151 residue on Keap1 [70]. Nrf2 is therefore free to translocate to the nucleus where it binds to ARE (Figure 10).

The activation of extracellular signal-regulated kinases ERK1/2 and Akt, also known as protein kinase B, was observed after GSGa treatment in both cMSC and NHDF (see also Figure S6a in the Supplementary Materials). ERK1/2, belonging to the Ras-Raf-MEK-ERK signal transduction cascade, are involved in the regulation of several cellular processes such as cell adhesion, cell cycle progression, cell migration, cell survival, differentiation, metabolism, proliferation and transcription [71]. The activation of ERK1/2 follows the phosphorylation of the residues Tyr 204/187 and Thr 202/185 by MEK [71]. ERK1/2 targets include hundreds of cytoplasmic or nuclear substrates involved in different cellular processes. ERK1/2 could be responsible for the pro-survival and anti-apoptotic effect of GSGa, considering that an increased activation of ERK1/2 (p-ERK1/2) was observed in treated cMSC compared to control. Akt activation has also been associated with numerous crucial cell functions, such as proliferation, differentiation, cell migration, survival and angiogenesis [72].

This marked activation of Akt was also in agreement with the effect induced by H_2_S-releasing agents on normal cells and BMSCs [45,73,74]. Therefore, our results are in agreement with recent findings that link H_2_S pro-survival effects to the activation of the PI3K/Akt pathway [72,75,76].

GSGa treatment of cMSCs also led to increased expression of the proteins α-smooth muscle actin (α-SMA) and Cx43. Their increased expression can be linked to a commitment of the cells towards a cardiac phenotype. Cx43, in fact, is the most abundant isoform of gap-junction channels in cardiac tissue and its increased expression is also in agreement with our previous data obtained using H_2_S- releasing nanoemulsions [40]. Generally, Cx43 expression is low in MSC [77], while high Cx43 expression in MSC can improve their survival and cardiomyogenesis after transplantation [78,79].

Thus, the therapeutic efficacy of cardiac progenitor/stem cell transplantation could be enhanced by the overexpression of Cx43 because it promotes neovascularization, reduces infarct size and preserves cardiac function preservation in the ischemic heart [80,81,82]. Cell therapy using MSC overexpressing Cx43 reduces infarct size, improving the heart functionality [74,80]. These results suggest a possible use of GSGa preconditioning of cMSC for improving their survival and eventually potentiate their ability to promote cardiac muscle tissue repair.

Thanks to the cytoprotective properties of H_2_S-donors, the preconditioning of stem cells before their transplantation has recently gained attention. H_2_S-donors are, without a doubt, promising exploitable tools to overcome the massive cell death that occurs after the implantation of stem cells in the site of injury for stem cell therapy. Currently, not many studies have investigated the use of H_2_S-donors in tissue repair, and most of them are focused on short-term preconditioning of the stem cells prior to transplantation. The time-span of the pretreatment varies from as short as 30 min to 48–72 h [45,81,82]. A selected cell line named GcMSC was obtained after long-term preconditioning and was here characterized. GcMSCs were more resistant to the oxidative stress and further addition of GSGa to the medium of the GcMSC culture, and led to a survival higher than that obtained with only the addition of GSGa without preconditioning. This result suggests that the combination of these two approaches might be the best choice for potential in vivo applications.

This increased resistance to the oxidative stress was also in agreement with higher levels of expression of proteins associated with pro-survival/proliferation, such as pERK-1, p-Akt, Bcl2 and Trx1, in GcMSC compared to those in cMSC (Figure 6e). One of the most important features of a successful stem-cell based therapy is the ability of the implanted cells to migrate to the site of injury, proliferate and eventually differentiate in order to replace the damaged tissue. The cell migration ability was tested using both the percentage of scratch wound closure and the trans-well migration, and in both cases, the GcMSC migration was significantly higher than that of cMSC.

These results are in agreement with the recent studies that demonstrate that H_2_S is able to promote migration and wound healing. The underlying mechanism involves increased levels of p-Akt, p-ERK1/2 and phosphorylated glycogen synthase kinase-3b [81,82,83]. Overall these results demonstrate that prolonged cell GSGa pre-conditioning could represent a powerful tool for selecting adult stem-cell lines that maintain an increased proliferative and migration capability and resistance to oxidative stress, suggesting a potential successful approach for future in vivo/therapeutic applications. GcMSC were able to differentiate into different cell types and the analyses performed did not show any relevant differences between the cell plasticity of GcMSC and cMSC.

The microarray data analysis showed that 52.5% of the genes, whose expression was altered upon the GSGa treatment, (+GSGA), were linked to the cardiovascular system/cardiovascular diseases, neurodegenerative diseases and diabetes. As previously stated, the study of the effects of H_2_S on pathological conditions is focused mainly on the field of cardiovascular and neurodegenerative diseases. The first report of an H_2_S cytoprotective effect goes back to 1996, when Abe and Kimura reported a beneficial role of NaHS in inducing long-term potentiation of the hippocampus at micromolar concentrations [84]. After that, interest gradually increased and other roles of this *gasotransmitter* in the central nervous system (CNS) and in neurodegenerative diseases were reported. In particular, H_2_S concentration is relatively high in the brain, due to the tissue-specific expression of CBS. Indeed, H_2_S has been reported to enhance N-methyl-D-aspartate (NMDA) receptor-mediated responses and to modulate Ca^2+^ and pH homeostasis in neurons, microglial cells and astrocytes [85]. Exogenous administration of H_2_S has been therefore seen as a potential therapeutic tool for the cure of several CNS diseases including Alzheimer’s disease, Parkinson’s disease, ischemic stroke and traumatic brain injury [86]. H_2_S also plays a central role in regulating cardiovascular system homeostasis. Changes in endogenous H_2_S levels have been correlated to many diseases, including heart failure, myocardial ischaemia and atherosclerosis [87]. On this basis, our results are in agreement with the effects of other H_2_S donors on cardiovascular and neurodegenerative diseases and suggest the possible value of investigating other potential target proteins whose expression can be regulated upon treatment with the natural H_2_S-donor GSGa. 

## 4. Materials and Methods 

### 4.1. Preparation of Water-Soluble Extracts from Allium sativum L. and H_2_S-Release Assay

The garlic water-soluble extracts (GaWS and GSGa) were prepared as previously described [16]. Briefly, 5 g of garlic cloves were crushed in 50 mM Tris-HCl buffer at pH 7.5 at room temperature for about 5–10 min with or without 10 mM reduced glutathione (GSH) and then the crushing procedure was continued in liquid N_2_. After centrifugation, the water soluble fraction was stored at −20 °C for molecular characterization by RP-HPLC. RP-HPLC analysis was performed using mod. LC-10AVP (Shimadzu, Milan, Italy), equipped with a UV detector (Shimadzu, Milan, Italy) and a C_18_ column (150 mm × 4.6 mm, 5 μm, CPS Analitica, Rome, Italy). The solvent B gradient (solvent B: 80% CH_3_CN, 0.1% TFA; solvent A: 0.1% TFA) used was: 0–5 min, 0%; 5–55 min, 60%; 55–60 min, 60% and 65–85 min 90%. The elution was monitored at 220 nm. To obtain the dry weight of the extract (and therefore its concentration), 100 μL of GSGa extract were lyophilized.

### 4.2. H_2_S Release Assay

H_2_S production and release by GSGa, GYY4137 and Na_2_S was assessed by methylene blue assay as previously described [16]. Briefly, each sample with 1 mM dithiothreitol (DTT) in 50 mM Tris HCl, pH 7.4 (150 µL final volume) was incubated at 37 °C on a shaker for 30 min. After the incubation, 20 µL of solution I (20 mM N’, N’-dimethyl-p-phenylene-diamine-dihydrochloride in 7.2 M HCl) and 20 µL of solution II (30 mM FeCl_3_ in 1.2 M HCl) were added to each solution. After an incubation time of 10 min at room temperature, coupled to gentle mixing of the solutions, absorbance was measured at 670 nm. Na_2_S was used to elaborate a standard curve (Figure S1A in Supplementary Materials) and the H_2_S- release from GYY4137 was also tested by MB assay and compared to that from GSGa (Figure S1B Supplementary Materials). All the results in this work were elaborated and plotted using GraphPad Prism version 5.0 software (GraphPad Software, San Diego, CA, USA).

### 4.3. Plasmid DNA Cleavage Inhibition Assay 

These experiments were performed as previously described with minor modifications [40]. Briefly, reagents were added to a 0.5 mL microfuge tube in the following order: 0.5 μg of plasmid DNA, 20 mM Tris-HCl steril pH 7.4 buffer, 10.8 mg/mL of GSGa, 100 μM of either H_2_O_2_ or CuCl_2_ or CoCl_2_ and 10 mM ascorbic acid, and sterilized ddH_2_O to a final volume of 10 μL. After an incubation period of 15 min at 37 °C, the reactions were stopped by the addition of 2 μL of gel loading buffer (5% glycerol, 0.125% bromophenol blue, 25 mM EDTA) and freezing. Samples were kept on ice until electrophoresis in 0.8% agarose gel in TAE buffer.

### 4.4. Cell Viability Assay

Cell viability and proliferation were tested either by MTT 3-(4,5-dimethylthiazol-2-yl)-2,5-diphenyltetrazolium bromide [88] or WST-1(4-[3-(4-lodophenyl)-2-(4-nitrophenyl)-2H-5-tetrazolio]-1,3-benzene disulfonate (Cell Proliferation Reagent WST-1, Roche, Mannheim, Germany) [88] assay as indicated. After each treatment, the medium was replaced with fresh DMEM high glucose without phenol-red (Gibco, Life Technologies, Milan, Italy) containing tetrazolium salt WST-1 (5% *v*/*v*) or MTT (0.5 mg/mL). The cells were then incubated for 3 h at 37 °C, 5% CO_2_. Absorbance of the medium was evaluated using a microplate reader at a wavelength of 450 nm for WST-1. For MTT assay, formazan crystals were solubilized with a solution of isopropanol and DMSO (1:1) and then the absorbance was measured at a wavelength of 570 nm.

### 4.5. Cell Migration

#### 4.5.1. Scratch Wound Healing Assay

cMSC were seeded into 24-well plates (6.5 × 10^4^ cells/cm^2^) and incubated over night at 37 °C, 5% CO_2_, so that the cells would reach confluency the next day. After 24 h, a scratch-wound was created with a 1 mL sterile pipette on the cell monolayer of each well. The medium was then removed and cells were washed twice with PBS; fresh medium was added to each well (800 μL/well). Area of the scratch-wound at time 0 and after 48 h was measured with ImageJ Software. Percentage of wound closure was measured as follows:

Wound closure (%) = (Wound surface area after 48 h/Wound surface area at time 0) × 100

#### 4.5.2. Trans-Well Migration Assay

Cell migration was assessed using 8 µm-pore-size Falcon ^TM^ Cell Culture Inserts (Thermo Fisher Scientific, Milan, Italy). cMSC (0.1 × 10^6^) were added on the upper chamber of the inserts in serum-free DMEM. Complete medium was added to the lower chamber of the inserts to attract the cells. After incubation for 6 h at 37 °C, 5% CO_2_, cells were removed from the upper surface of the trans-well membrane with a water-wetted cotton swab. Cells that had migrated on the other side of the membrane were fixed and stained for 20 min with a solution containing 6% (*v*/*v*) glutaraldehyde and 0.5% (*w*/*v*) crystal violet in deionized water. The inserts were then washed repeatedly with water. Air-dried membranes were analyzed by optical microscopy. 

### 4.6. Protection from Oxidative Stress 

NHDF were seeded at a density of 3 × 10^3^ cells/cm^2^ and cultured for three days in the absence or in the presence of 680 μg/mL of GSGa. After that, the cells were reseeded at a density of 10^4^ cells/cm^2^ and after 6 h of incubation, the medium was replaced with fresh medium containing 100 μM of H_2_O_2_. After 12 h, cell survival was assessed by optical microscopy. Two different experiments were performed in order to analyze the anti-oxidant effect of GSGa treatment on cMSC. In the first one, cMSCs and GcMSCs were seeded at a density of 5 × 10^3^ cells/cm^2^ and after 24 h of growth the medium was replaced with fresh medium containing 100 μM of H_2_O_2_ with or without the addition of 680 μg/mL of GSGa, GSH (100 μM), Na_2_S (100 μM) or GYY4137 (300 μM). After 24h, the medium was replaced with fresh DMEM medium (Gibco, Life Technologies, Milan, Italy) without phenol red and cell viability was assessed by WST-1 assay (Sigma-Aldrich, Milan, Italy). Cell cycle distribution analysis was performed by flow cytometry (FACS analysis) after 12 h of incubation. Briefly, cells (about 0.5 × 10^6^) were harvested and stained with 50 μg/mL propidium iodide (Sigma-Aldrich, Milan, Italy) for 30 min at 4 °C. After incubation, the samples were immediately analyzed using a FACSCalibur flow cytometer (Beckton and Dickinson, San Josè, CA, USA). The data obtained were then elaborated with the WinMDI free software. The second experiment was performed using cMSC and GcMSC that were seeded at a density of 10^4^ cells/cm^2^ and after 6 h of growth, the medium was replaced with fresh medium containing 100 μM of H_2_O_2_. After 12 h of growth, cell survival was assessed by optical microscopy.

### 4.7. cMSC and NHDF Cultures and Immunofluorescence Analyses 

Cell studies were conducted on cMCS and NHDF (Lonza, Basel, Switzerland) cell lines. cMSC were extracted by auricular biopsies made during the course of coronary artery bypass surgery from patients after signing a written consent form as previously described [38,39,40,89,90]. Cell cultures were grown in DMEM (Dulbecco’s modified Eagle medium) (Gibco, Life Technologies, Milan, Italy), containing 10% *v/v* FBS (Fetal Bovine Serum) (Gibco, Life Technologies, Milan, Italy), 1% *w/v* penicillin-streptomycin (Sigma-Aldrich, Italy), and 1% *w/v* L-glutamine (Sigma-Aldrich, Italy). To perform the microscopy analyses, cMSCs, after the treatment, were washed in PBS, fixed in PBS with 4% *v/v* PFA at 4 °C for 15 min, permeabilized with 0.2% *v/v* Triton X-100 (Sigma-Aldrich, Italy) for 30 min and after washing, were incubated with specific antibodies for immunofluorescence microscopy. The antibodies used were anti-α-smooth muscle actin (α-sma) mouse, anti-connexin-43 (Cx43) mouse (Sigma-Aldrich, Italy), anti-human troponin T2 (TNNT2), followed by the appropriate Alexa Fluor^®®^ 488 fluorochrome-conjugated secondary antibody (Invitrogen, Life Technologies, Milan, Italy). Nuclei were stained with Hoechst 33342 (Sigma-Aldrich, Italy). The cells were analyzed by fluorescence microscopy using a Nikon Filter microscope and Lucia G version 4.61 software.

### 4.8. Protein Extraction and Western Blot Analysis

Proteins were extracted from cMSCs using 100 μL of RIPA buffer containing a protease inhibitor cocktail (Sigma-Aldrich, Italy) and pervanadate (Sigma-Aldrich, Italy) as phosphatase inhibitor and after 90 min of incubation in ice were sonicated for 10 sec at 0 °C. Samples were centrifuged for 10 min at 8000 rpm at 4 °C. Protein content was determined by BCA protein assay (Sigma-Aldrich, Milan, Italy), and the SDS-PAGE of cell extracts (30 μg of protein) were performed using 12 or 15% polyacrylamide gel. PVDF membranes (Sigma-Aldrich, Italy) were used for electro-blotting and were then blocked and probed with primary monoclonal antibodies (Ab-ERK1/2 rabbit, Ab-p-ERK1/2 rabbit, Ab-Trx rabbit, Ab-NQO1 rabbit, Ab-Akt and Ab-pAKT-(pSer473) rabbit, A, Ab-α-sma rabbit, b-Cx43 rabbit) (Sigma-Aldrich, Italy,) overnight at 4 °C. Immunoblots were next processed with secondary antibodies (Sigma-Aldrich, Italy) for 2 h at room temperature. Immunoblot with Ab-GAPDH rabbit or Ab-β-tubulin mouse (Sigma-Aldrich Italia, Milan Italy) were also probed for controlling the protein loading. The protein complex formed upon incubation with specific secondary antibodies (dilution 1:10000) (Sigma-Aldrich, Milan, Italy). Immunoblots were probed with a Super Signal West Pico kit (Thermo Scientific, Milan, Italy) to visualize signal, followed by exposure to Fluorchem Imaging system (Alpha Innotech Corporation-Analitica De Mori, Milan, Italy) or using a X-ray film (Kodak, Sigma-Aldrich, Italy).

### 4.9. Cell Differentiation

Control untreated cMSC and those pre-treated for one month with 140 μg/mL of GSGa (GcMSCs) were seeded in 35 mm dishes at a density of 5000 cells/cm^2^ until they reached a confluency of 80–90%. For adipogenic and osteogenic differentiation, the cells were stimulated for 3 days in the differentiation mediums, StemPro^®®^ Adipogenesis Differentiation Kit and StemPro^®®^ Chondrogenesis Differentiation Kit (Gibco, Life Technologies, Milan, Italy), respectively. After 3 days, the medium was removed and the cells were washed twice with PBS and fixed with 4% *v/v* PF for 20 min at room temperature in the dark. Cells were then washed once with PBS, stained with Alcian Blue pH 2.5 to detect chondrogenic differentiation or Adipo Red for the adipogenic differentiation and analyzed by optical and fluorescent microscopy, respectively. For osteogenic differentiation, confluent cells were stimulated for 3 days with DMEM supplemented with 10% FBS, 1% *w/v* penicillin-streptomycin (Sigma-Aldrich, Italy), 1% *w/v* L-glutamine (GIBCO, Life Technologies, Milan, Italy), 1% *v/v* non-essential amino acids solution (Sigma-Aldrich, Italy), 50μg/mL ascorbic acid, 10 mM β-glycerophosphate and 10 nM dexamethasone. After the stimulation period, the cells were stained with Alizarin Red dye and analyzed by optical microscopy. Cardiogenic differentiation was performed following the instructions provided with the Human Cardiomyocyte Immunocytochemistry Kit^®®^ (Life Technologies, Milan, Italy); after differentiation, cells were fixed as previously described and stained with a primary mouse anti-human troponin T2 (TNNT2) antibody and an Alexa Fluor^®®^ 488 donkey anti-mouse secondary antibody.

### 4.10. Microarray 

RNA was extracted from cells, using a RNAeasy Kit (Qiagen, Manchester, UK). RNA was reverse transcribed, converted to cDNA, amplified, and labeled with a cyanine-3 dye using a Low Input Quick Amp labeling kit from Agilent. Labeled cRNAs were hybridized to human gene expression microarrays (Agilent, Cheshire, UK). The data were extracted using the Agilent Feature Extraction software (version 10.7.3.1) and analyzed using Agilent GeneSpring GX software (version 12.1, Agilent, Cheshire, UK). An unpaired Student’s t test with Benjamini–Hochberg multiple testing correction was applied in order to analyze significant differences of expression, mRNAs with a *p* value of less than or equal to 0.05 and a fold change of greater than 1.5 were considered to be both statistically significant. Heat-maps were generated by Agilent GeneSpring GX software (version 12.1) with a hierarchical clustering algorithm based on normalized intensity value by using Euclidean similarity measurements (cut-off *p* value 0.05; cut-off fold change 1.5). Microarray analyses were performed using the DAVID 6.8 program [91,92] and InteractiVenn webtool (http://www.interactivenn.net).

### 4.11. Statistical Analysis 

GraphPad Prism version 6.0 for Windows (GraphPad Software, San Diego, CA, USA) was used for the statistical analysis. Data obtained from three or five independent experiments were quantified and analyzed for each variable using a one-way ANOVA test or in some cases one-tailed Student’s t-test. A *p* value of < 0.05 was considered to be statistically significant. Standard deviations or the standard error means were calculated and presented for each experiment.

## 5. Conclusions

The endogenous H_2_S levels help regulating the equilibrium of several organs, including the respiratory, reproductive, neuronal, renal, cardiovascular, gastrointestinal and liver systems. The broad physiological role of this lipid-soluble gasotransmitter is due to its membrane permeability, although its unique chemical reactivity towards some macromolecules in different cell lines makes this gas a selective signalling molecule. Here a garlic water-soluble extract obtained with glutathione conjugation was used as a natural H_2_S-releasing agent to analyse the effects of both acute and prolonged preconditioning on progenitor stem cells. Although the protective effect of H_2_S by the oxidative stress was well investigated in the human cells using NaHS as H_2_S donor [1,41], the effects of potential nutraceutical bio-products, such as GSGa, on MSC has not yet been assessed. Several studies suggest that the inherent reparative capability of the body could in theory be supported by incrementing the efficiency of the endogenous MSC via therapeutic exogenous MSC [93,94,95]. 

One of the most relevant problems in cell-based therapy is the optimization of the stem cell delivery system and of the capability of multipotent stem cells to proliferate, migrate and differentiate, generally, in compromised sites where active oxidative and inflammatory processes are ongoing, with the aim of improving tissue repair and regeneration.

We demonstrated that the cell line selection by GSGa conditioning significantly improves the ability of the cMSC to proliferate, migrate and survive oxidative injury by activation of the expression of both HRE- and ARE-mediated transcription genes. The subsequent increase in antioxidant enzymes and molecules might protect against cellular senescence induced by oxidative stress. Moreover, we demonstrated that the prolonged GSGa treatment does not affect the cell plasticity of cMSC, although it improves the expression of proteins such as α-SMA and Cx43, which are important in muscle tissue commitment. The results presented here suggest the possibility of ameliorating MSC therapy by prolonged treatment with natural H_2_S-releasing donors that could improve MSC homing to the site of injury, promoting their cell proliferation, migration and survival under oxidative stress conditions and ultimately favouring the capability of MSC to secrete paracrine factors with both immunoregulatory and structural functions for microenvironment regeneration.

## Supplementary Materials

Supplementary Materials can be found at https://www.mdpi.com/1422-0067/21/5/1638/s1.

## Abbreviations

3-MTS	3-mercapto-piruvate sulfurtrasferase;
α-SMA	α-smooth muscle actin;
AcH3	acetylated histone H3;
ACTN2	Actinin alpha 2;
Akt	Protein kinase B;
ARE	Antioxidant response element;
BAD-NE	BSA/ALA/DADS nanoemulsion;
BCA	bicinchoninic acid;
BMSCs	bone marrow mesenchymal stem cells;
CBS	cystathionine β-synthase;
CNS	central nervous system;
CSE	cystathionine γ–lyase;
Cx43	connexin-43;
cMSCs	Lin^-^Sca-1^+^ human cardiac progenitor cells;
DADS	diallyl-disulfide;
DMEM	Dulbecco’s modified Eagle medium;
DATS	diallyl-trisulfide;
DU145	cells prostate cancer cell line;
ERK1/2	extracellular signal–regulated kinases 1/2;
FBS	Fetal Bovine Serum; H_2_S, hydrogen sulfide;
HO1	heme oxygenase 1
Keep1	Kelch ECH associating protein 1;
MEF2C	myocyte enhancer factor 2C
MSCs	mesenchymal stem cells;
NHDF	normal human dermal fibroblasts;
NQO1	NAD(P)H quinoneoxidoreductase 1;
NMDA	N-methyl-D-aspartate;
Nrf2	nuclear factor erythroid 2-related factor 2;
NSAIDs	non-steroidal anti-inflammatory drugs;
OSCs	Organo-sulfur compounds;
p21	cyclin-dependent kinase inhibitor 1;
SGCD	sarcoglycan delta;
Trx1	thioredoxin 1;
TYRO3	tyrosine-protein kinase receptor;
SRFBP1	serum response factor binding protein 1;
TFA	trifluoroacetic acid;
TNNT2	human Troponin T2;
TST	thiosulfate: cyanide sulfurtransferase enzyme.
