# Supplementary material for: Glutathione–Allylsulfur Conjugates as Mesenchymal Stem Cells Stimulating Agents for Potential Applications in Tissue Repair
Source: Int J Mol Sci. 2020 Feb 28;21(5):1638. doi: 10.3390/ijms21051638 (PMC7084915; doi:10.3390/ijms21051638)
Supplement: Supplementary file 1 [file ijms-21-01638-s001.pdf]

Supplementary Materials

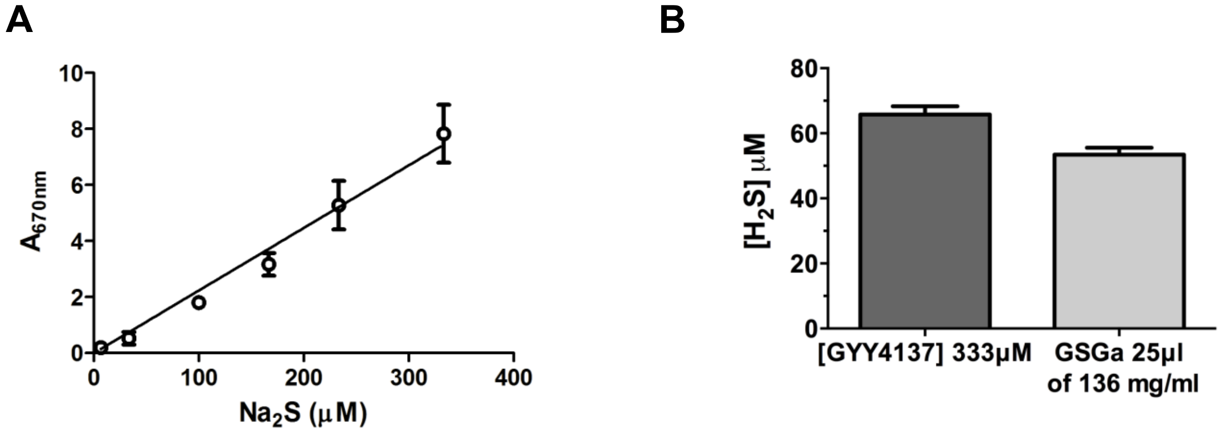

Figure S1. Calibration curve of H_2S releasing. **A**) Calibration curve of H_2S release by different concentrations of Na_2S. **B**) Amount (μM) of H_2S released by 333 μM GYY4137 and 25 μL of GSGa (136 mg/mL); measured by MB assay and spectrophotometric analysis.

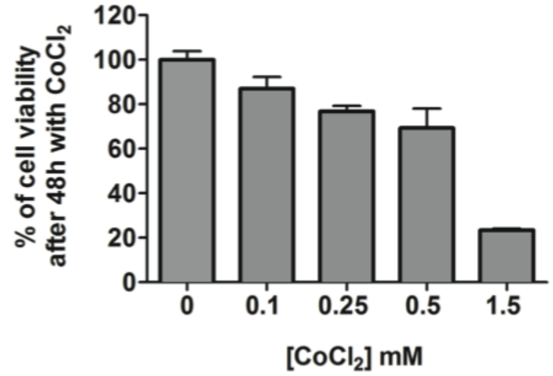

Figure S2. Cell viability of cMSC by MTT assay, after 48h of treatment with increased concentrations of $CoCl_2$.

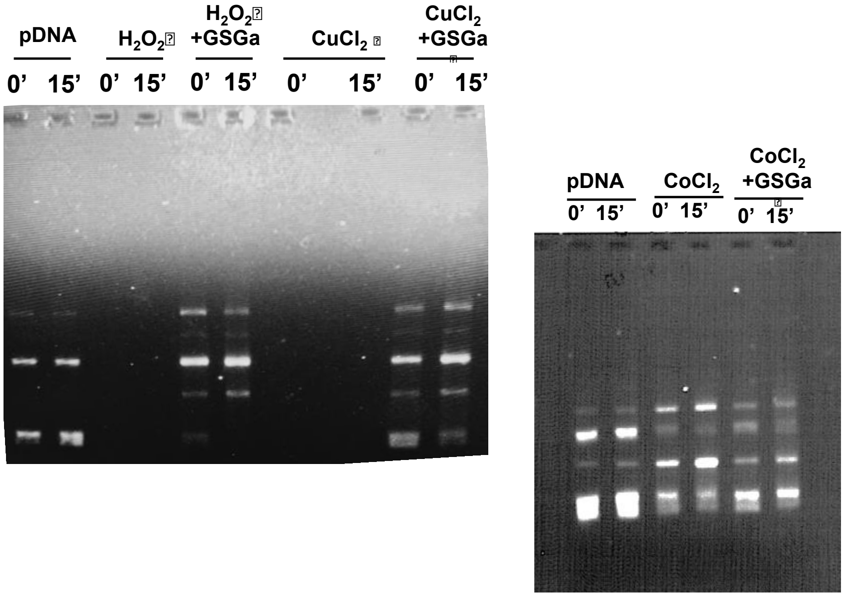

Figure S3. Integral images of the agarose gel elettrophoresis of pDNA incubated with H_2O_2, $CuCl_2$ or $CoCl_2$ in the presence and in the absence of GSGa corresponding to the Figure 2a,b.

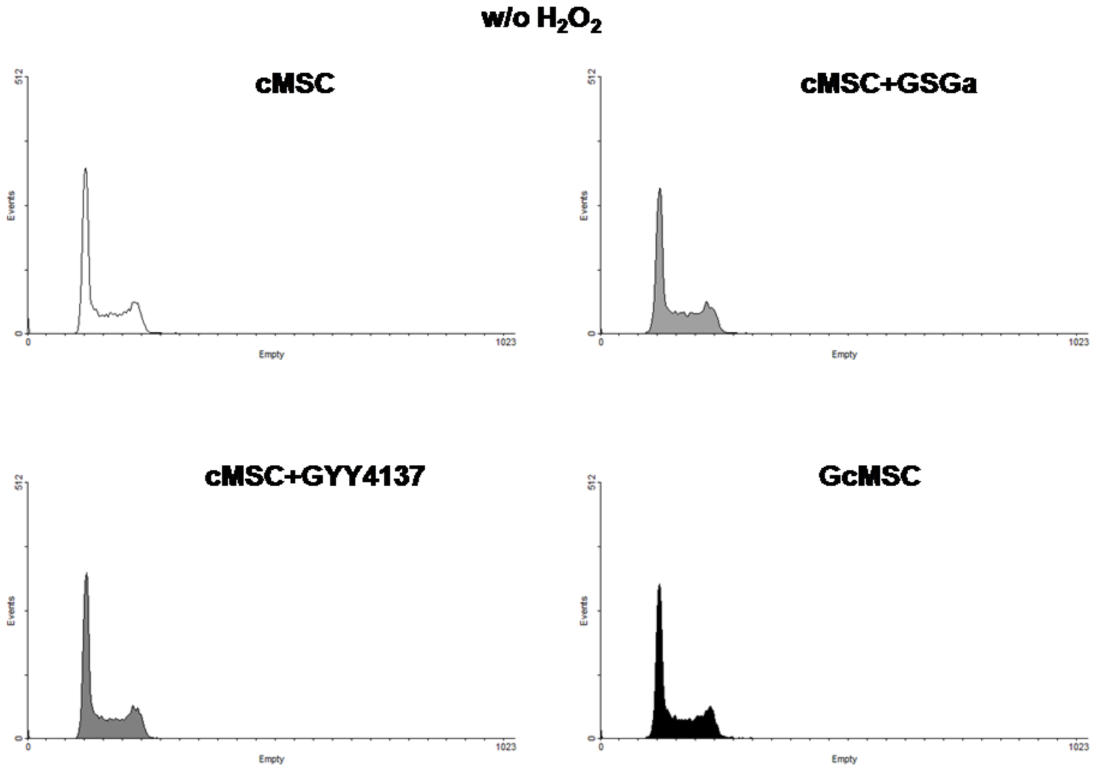

Figure S4. FACS cell cycle analysis of cMSC, cMSC growth in the presence of GSGa (680 μg/mL) (cMSC+GSGa) or of GYY4137 (80 mM) (cMSC+GYY4137) and GcMSC cultured for 24 h in the absence of H_2O_2.

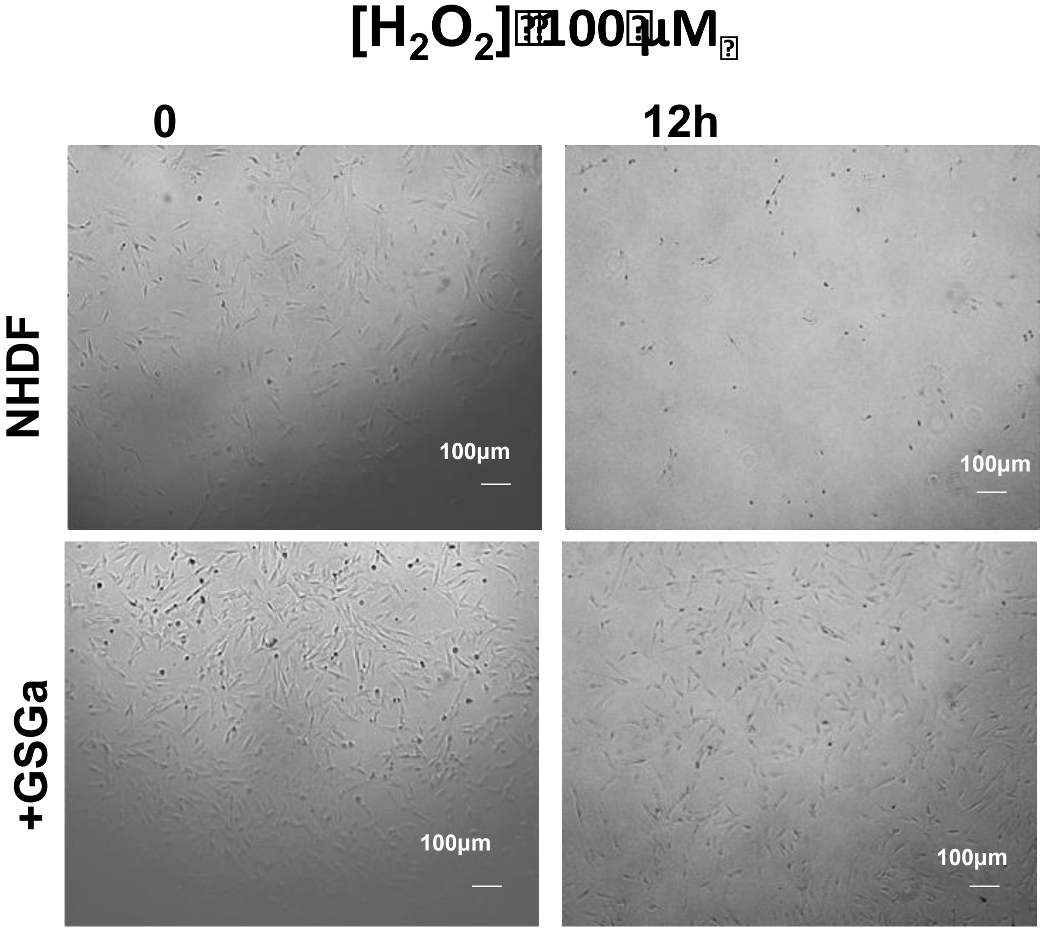

Figure S5. Magnification of the micrographs present in Figure 3a. Micrographs of NHDF after 0 and 12 h of treatment with H_2O_2 (100 µM), with (+GSGa) or without (NHDF) the addition of 680 µg/mL GSGa.

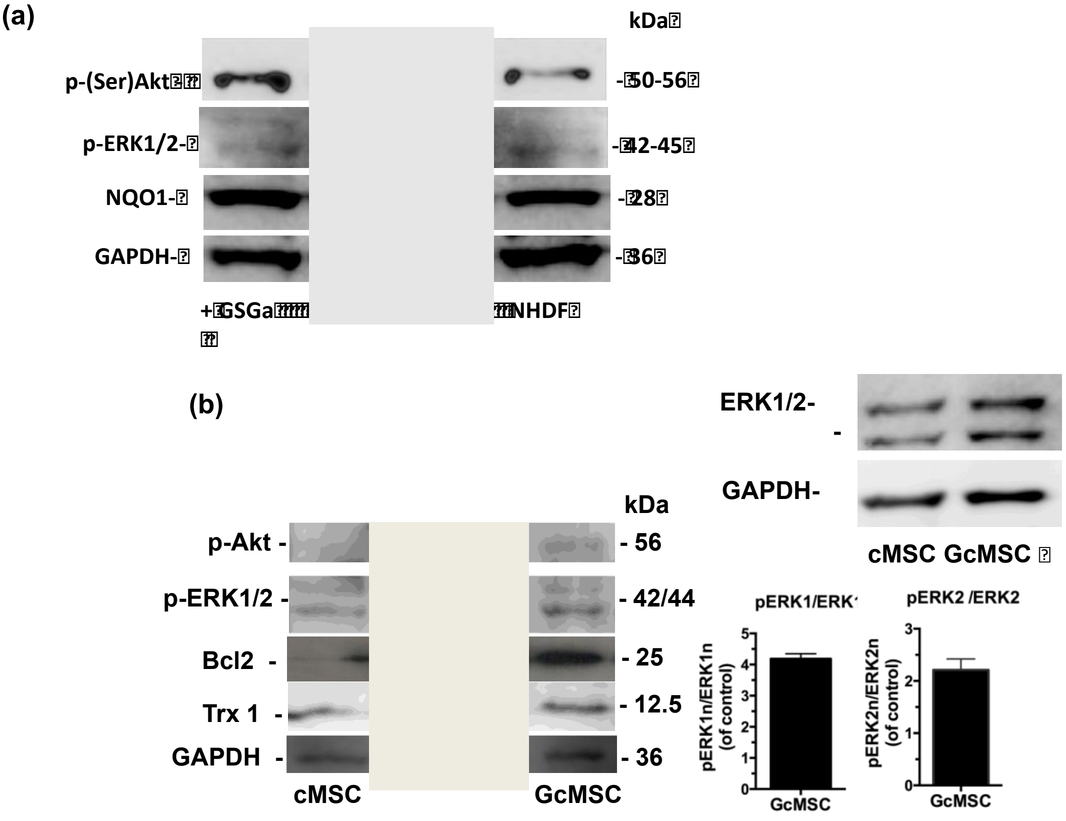

Figure S6. (**a**) Representative western blot analysis of the expression of p(Ser 473)Akt, p-ERK1/2, Bcl2, NQO1 in NHDF; (**b**) Representative western blot analysis of the expression of p(Ser 473)Akt, p-ERK1/2, Bcl2, Trx1 in cMSC and GcMSC.

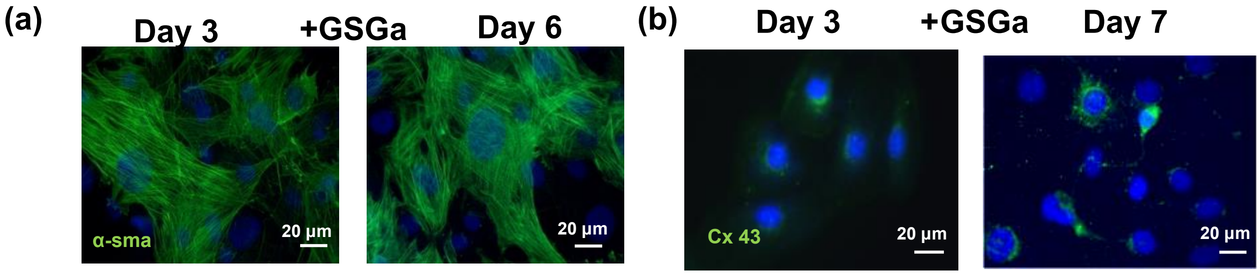

Figure S7. (**a**) α-SMA expression at 3 and 6 days is shown in green and (**b**) Cx43 at 3 and 7 days is stained in green, the nuclei are stained with Hoechst 33342.

Volcano Plots

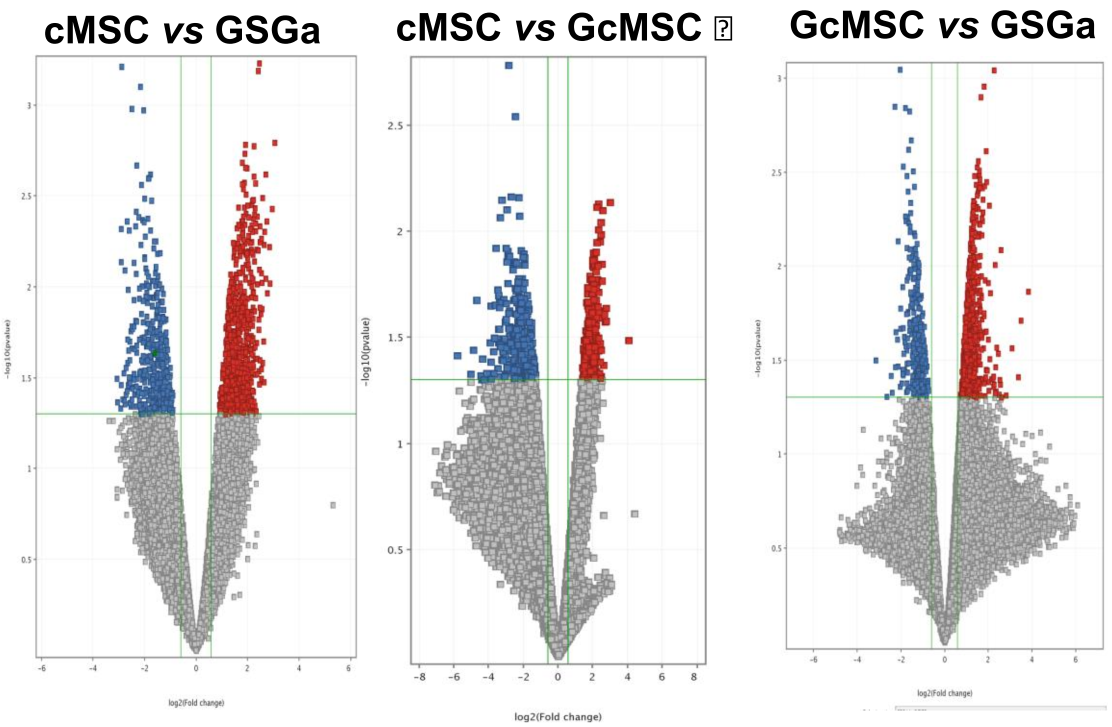

a)

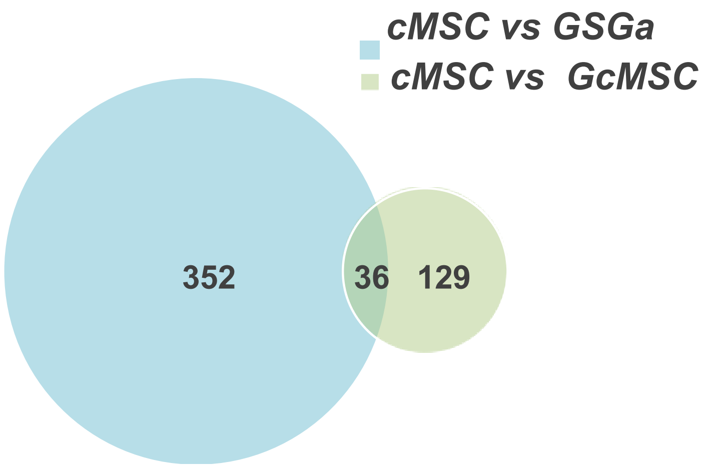

b)

Figure S8. Microarray analysis. **a**) Volcano plots from microarray analysis. Volcano plots from transcriptional profiling analysis of cMSC following acute (GSGa) (3 days, left panel) or prolonged (right panel) (GcMSC) treatment with GSGa indicate variation of expression of analyzed genes. Bleu and red dots indicate respectively significantly down-regulated and up-regulated genes. *p* value cut-off 0.05; fold change cut-off 1.5. **b**) Venn Diagram depicts overlap of differentially regulated genes after 3 days (GSGa) and 30 days (GcMSC) of treatment. The diagram has been generated by using InteractiVenn webtool (http://www.interactivenn.net) inputting the probe name lists from the gene array data.

Table S1. Analysis by David 6.8 of the genes that are changed in both the GSGa treatments.

ID From	To	Species	Gene Name
4208	781152	Homo sapiens	myocyte enhancer factor 2C
387036	812157	Homo sapiens	glucuronidase, beta-like 1
3115	783974	Homo sapiens	major histocompatibility complex, class II, DP beta 1
1572	780823	Homo sapiens	cytochrome P450, family 2,subfamily F, polypeptide 1
100170226	789502	Homo sapiens	small ILF3/NF90-associated RNA C3
2906	786599	Homo sapiens	glutamate receptor, ionotropic, N-methyl D-aspartate 2D
187	805439	Homo sapiens	apelin receptor
79843	794370	Homo sapiens	family with sequence similarity 124B
387111	811718	Homo sapiens	chromosome 6 open reading frame 181
133522	810592	Homo sapiens	peroxisome proliferator-activated receptor gamma, coactivator 1 beta
115350	788996	Homo sapiens	Fc receptor-like 1
6521	791678	Homo sapiens	solute carrier family 4, anion exchanger, member 1 (erythrocyte membrane protein

			band 3, Diego blood group)
153443	811107	Homo sapiens	serum response factor binding protein 1
3769	806972	Homo sapiens	potassium inwardly-rectifying channel,subfamily J, member 13
64320	772726	Homo sapiens	ring finger protein 25
57282	789195	Homo sapiens	solute carrier family 4, sodium bicarbonate transporter, member 10
89866	791541	Homo sapiens	SEC16 homolog B (*S. cerevisiae*)
83549	781739	Homo sapiens	uridine-cytidine kinase 1
23070	792478	Homo sapiens	FtsJ methyltransferase domain containing 2
407006	772175	Homo sapiens	microRNA 221
85004	776084	Homo sapiens	RAS-like, estrogen-regulated,growth inhibitor
440131	792858	Homo sapiens	similar to bA90M5.1 (novel protein)
**ID**	**Gene Name**	**Species**	**COG_ONTOLOGY**
1572	cytochrome P450, family 2, subfamily F, polypeptide 1	Homo sapiens	Secondary metabolites biosynthesis, transport, and catabolism
4208	myocyte enhancer factor 2C	Homo sapiens	Transcription
83549	uridine-cytidine kinase 1	Homo sapiens	Nucleotide transport and metabolism
**ID**	**Gene Name**	**Species**	**KEGG_PATHWAY**
187	apelin receptor	Homo sapiens	Neuroactive ligand-receptor interaction
1572	cytochrome P450 family 2, subfamily F, polypeptide 1	Homo sapiens	Metabolism of xenobiotics by cytochrome P450, signaling pathway
2906	glutamate receptor, ionotropic, N-methyl D-aspartate 2D	Homo sapiens	Neuroactive ligand-receptor interaction
3115	major histocompatibility complex, class II, DP beta 1 hsa04514:Cell adhesion molecules (CAMs)	Homo sapiens	Antigen processing and presentation
4208	myocyte	Homo	MAPK signaling pathway

	enhancer factor 2C	sapiens	
3549	uridine-cytidine kinase 1	Homo sapiens	Pyrimidine metabolism Drug metabolism

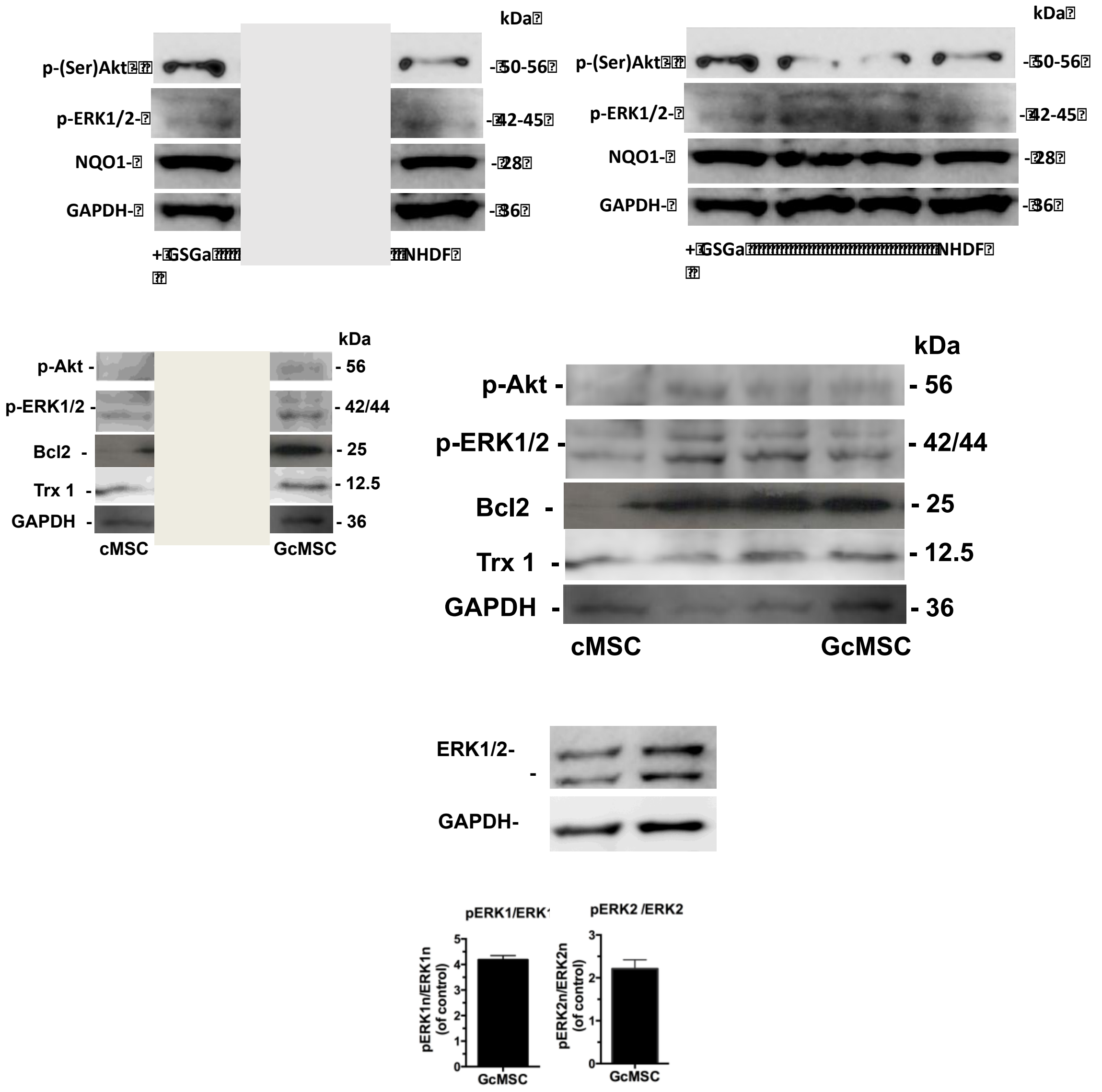

Figure S9. Original figures of the western blottings.

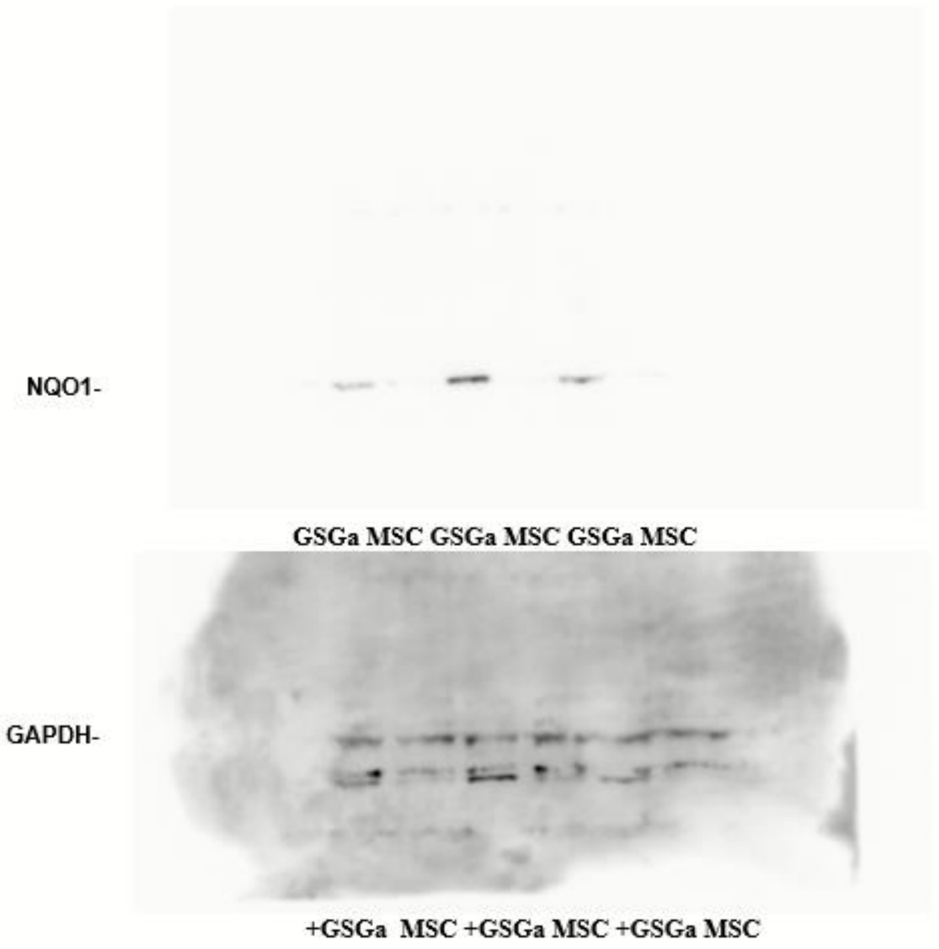

Figure S10. Original western blot of figure 4B that show the expression of NQO1 in MSC w/o treatment with GSGa.
